# Integrated single‐cell RNA sequencing and spatial transcriptomics analysis reveals the tumour microenvironment in patients with endometrial cancer responding to anti‐PD‐1 treatment

**DOI:** 10.1002/ctm2.1668

**Published:** 2024-04-22

**Authors:** Junfeng Chen, Yunfeng Song, Jian Huang, Xiaoping Wan, Yiran Li

**Affiliations:** ^1^ Department of Shanghai Key Laboratory of Maternal Fetal Medicine, Shanghai First Maternity and Infant Hospital, School of Medicine Tongji University Shanghai China; ^2^ Department of Gynecology, Shanghai First Maternity and Infant Hospital, School of Medicine Tongji University Shanghai China

Dear Editor:

The response of patients with endometrial cancer (EC) to anti‐PD‐1 therapy varies.^1^ Single‐cell RNA sequencing (scRNA‐seq) and spatial transcriptomics analysis were used for identifying specific immune cell populations that might exert a vital role in determining the sensitivity to anti‐PD‐1 therapy in EC. The quantities and activities of CD8 cytotoxic cells and Tregs were abnormal in non‐responders to anti‐PD‐1 therapy according to the obtained findings. Additionally, this study determined several ligand–receptor interactions associated with T‐cell dysfunction. Further exploration of distinct T‐cell subgroups may help address differences between patients responsive and non‐responsive to anti‐PD‐1 therapy in EC.^2^

Figure 1 illustrates the specific treatment plan for each patient and the changes in imaging before and after the treatment. We performed scRNA‐seq and spatial transcriptomics analysis on samples from Cases A and B (Table S1 and Figure 2A). A total of 15 150 cells were categorised into nine cell types based on marker gene expression in the samples (Figure S1A,B). We constructed a Uniform Manifold Approximation and Projection (UMAP) diagram that incorporates the expression profiles of the selected marker genes (Figure 2B). Figure S1C displays the top 10 differentially expressed genes for each of these nine major cell types. Our investigation revealed that T cells were the predominant type of immune cells (Figure 2C,D). The exact number of each cell type is presented in Figure 2D. We performed multiplex immunofluorescence (mIF), and the expression of immune cells in Case A was higher than that in Case B (Figure 2E). To characterise cancer cells, we examined large‐scale variations in the chromosomal copy number variations within each cell type.^3^ Ultimately, we classified all cell types, except for epithelial cells, as normal. Compared with these normal cells, a subset of epithelial cells exhibited substantial variations in their copy numbers (Figure S1D,E).

**FIGURE 1:**
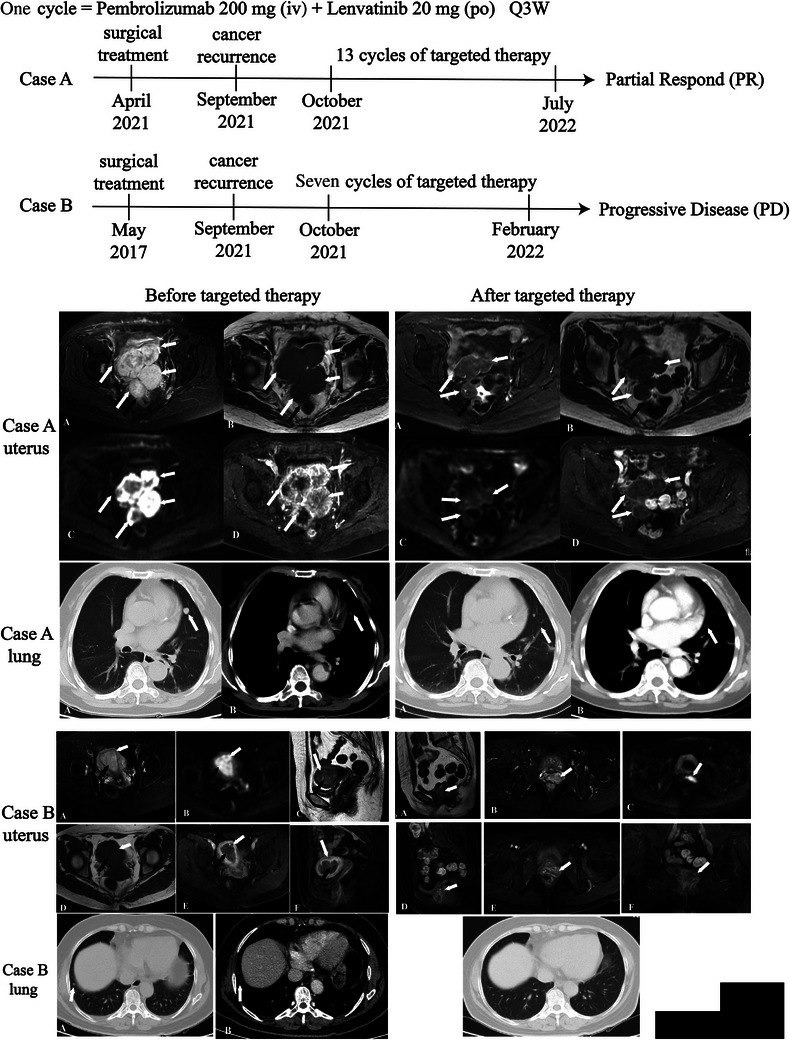
Patient with treatment plan and computed tomography (CT), magnetic resonance imaging (MRI) changes before and after treatment used in the study.

**FIGURE 2:**
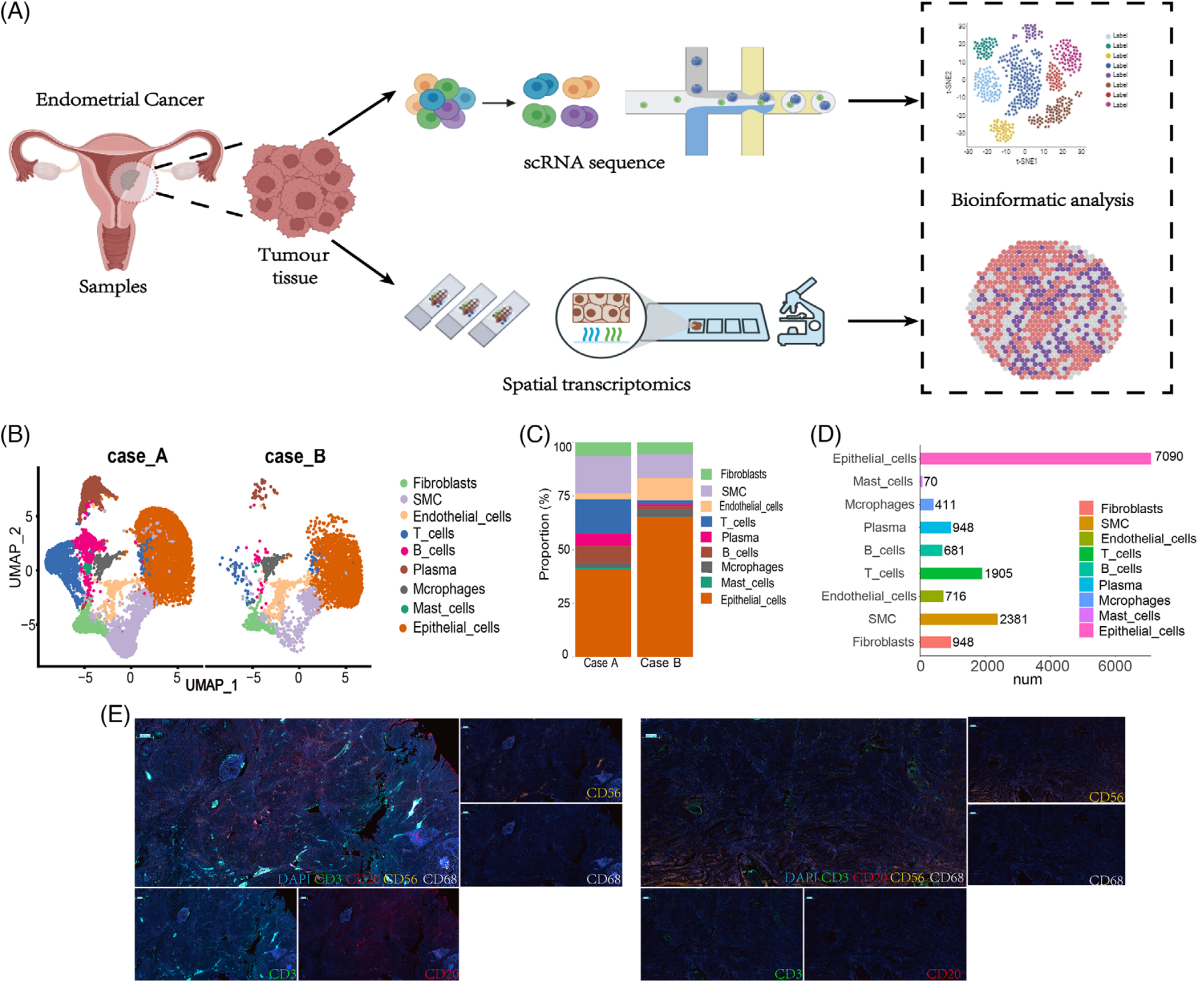
Diverse cell types from Case A and Case B delineated by single‐cell transcriptomic analysis. (A) Workflow of single‐cell RNA sequencing (scRNA‐seq) and spatial transcriptome analyses applied to endometrial cancer tissues. (B) Distribution of each cell type from Case A (left) and Case B (right) is displayed. (C) The proportion of different cell types in two clinical groups. (D) All cell numbers of each cell type in this analysis are summarised. (E) Representative multiplex immunofluorescence of tumour immune cell markers in Case A and Case B. T cells: CD3 (green), B cells: CD20 (red), macrophages: CD68 (white), NK cells: CD56 (orange), cell nucleus: diamidino‐phenyl‐indole (DAPI) (blue). Scale bar = 500 µm.

Considering the critical role of anti‐PD‐1/PD‐L1 in combating tumours, drugs targeting this pathway can alleviate T‐cell suppression, thereby reinvigorating their ability to target and eliminate malignant cells.^4^ These NK/T cells were divided into five subclusters based on UMAP analysis (Figure S1F,G). We examined the top 10 differentially expressed genes within each T subgroup (Figure S1H). We systematically examined five T‐cell subpopulations to assess both the quantity and genetic characteristics of each cell subtype (Figure 3A). CD8^+^ cytotoxic cells and Treg cells were expressed only in Case A (Figure 3B,C). Differentially expressed genes in T cells are presented in the heatmap and volcano plot (Figures S1I,J). Gene set variation analysis (GSVA) provides functional annotations for the four T subgroups (Figure 3D). The GSVA results for CD8^+^ cytotoxic cells and Treg cells indicated gene enrichment in the cytokine–cytokine receptor interaction (Figure 3E,F). CellphoneDB analysis reveals intercellular communication among all cell types (Figure S1K,L) and identifies unique cellular crosstalk patterns. The frequency of receptor–ligand interactions was higher in Case A than in Case B (Figure 3G,H). Furthermore, CD74–MIF, CD74–COPA and CD74–APP pairs might mediate the association among CD8^+^ cytotoxic cells, Treg cells and other cells in responsive patients (Figure 3I). Studies have indicated that the CD74–MIF complex can regulate tumour immune escape.^5^ The Single‐cell regulatory network inference and clustering (SCENIC) analysis showed Module 1, encompassing the TFs SPI1, STAT1, ETS1, IKZF1 and IRF8, exhibited the most pronounced regulatory activity across different T‐cell subtypes (Figure 3J,K). In addition, the regulon activity score confirmed the upregulation of the majority of TFs such as SPI1, STAT1, ETS1, IKZF1 and IRF8 (Figure 3L).

**FIGURE 3:**
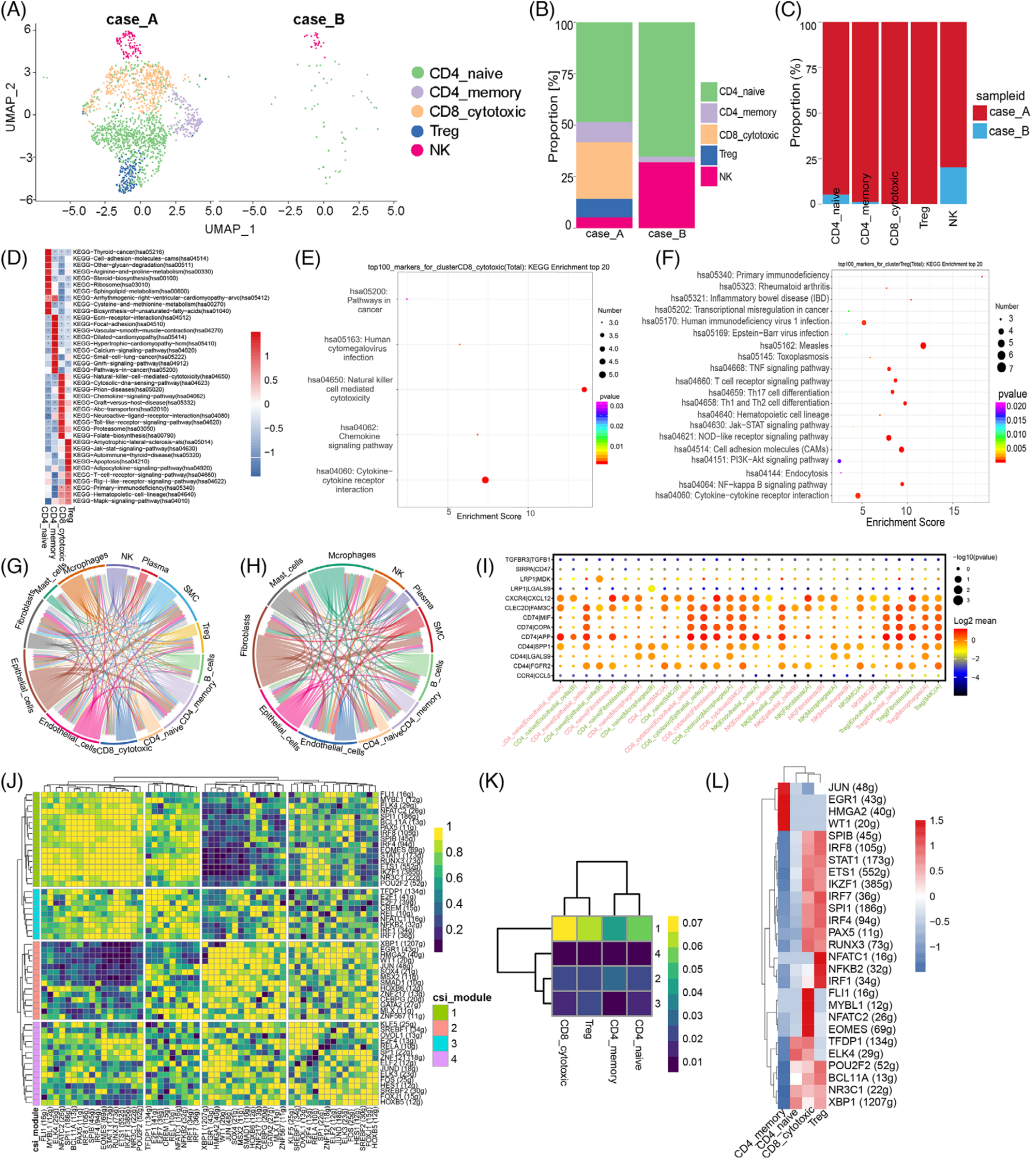
Distinct characteristics of T‐cell subtypes. (A) The cell distribution of each cell type of T/NK from Case A (left) and Case B (right) is displayed. (B, C) Histogram of T‐cell subtype compositions in the Case A (B) and Case B (C). (D) Gene set variation analysis (GSVA) indicates enriched pathways of each subset of T cells. (E, F) Kyoto Encyclopedia of Genes and Genomes (KEGG) analysis of top 100 genes in CD8 cytotoxic cells (E) and Treg cells (F). (G, H) The circle diagram illustrates the frequency of interactions among various cell types from Case A (G) and Case B (H). Arrow direction indicates the flow of interaction signals from ligand cells to receptor cells. Line thickness reflecting the abundance of ligand–receptor interaction pairs. (I) Ligand–receptor interaction pairs among Treg cells, CD8 cytotoxic cells and other cells in Case A and Case B. (J, K) The connection specificity index (CSI) matrix effectively showcases the correlation between regulons across various T‐cell subtypes. Through hierarchical clustering of these regulons, we can delineate four clearly defined regulon modules (J). To provide a visual representation of the regulatory activity within each module, a heatmap has been generated (K). The colour spectrum employed in the heatmap, ranging from blue to yellow, intuitively conveys the levels of activity, spanning from low to high. (L) The heatmap delineating the distribution of transcription factors among four distinct T‐cell subtypes. Parenthetical numerals correspond to the number of regulons associated with each transcription factor.

To confirm our single‐cell findings, we employed a spatial transcriptomic technique (Slide‐seqV2).^6^ Then, we assessed the spatial distribution of all cell types in Cases A and B to investigate their respective functions within the Tumor Micro Environment (TME) (Figure S2A,B). CD8^+^ cytotoxic cells and Treg cells were more enriched in Case A than in Case B (Figure 4A–C). Haematoxylin and eosin staining was carried out to distinguish between the tumour and vessel regions.^7^ Additionally, the gene expression characteristics of each specimen were analysed to further differentiate these areas by mapping scRNA data to Spatial Transcriptomics (ST) slides.^7^ Case A tumour tissues were enriched in the ferroptosis pathways and the P53 signalling pathway (Figure S2C,D). Simultaneously, the differentially expressed genes in Case A vessel tissues were enriched in the P53 pathway and the Extracellular Matrix (ECM)–receptor interaction (Figure S2E,F). Using unbiased clustering of ST sequencing data and spot features, we classified the spots into 13 clusters with the different gene expression profile of Case A differing from that of Case B (Figure 4D,E). We examined cell–cell communication within the TME and found that the interaction was stronger in Case A than in Case B (Figure 4F,G). To accurately show the spatial organisation of cell types without bias, we conducted robust cell type decomposition analysis.^8^ After deconvolution, we identified two spot patterns that were superimposed onto the original tissue region to visualise their spatial distribution (Figure 4H,I). Consistently, the interaction in Case A was stronger than that in Case B (Figures 4J,K and S2G,H). Furthermore, ligand–receptor interaction analysis revealed that CD74–MIF, CD74–COPA and CD74–APP ligand–receptor pairs might mediate the association among CD8^+^ cytotoxic cells, Treg cells and other cells (Figure 4L). As anticipated, although numerous ligand–receptor pairs were shared between both patients, certain pairs exhibited distinct interaction strengths among different cell subclusters. This highlights the presence of intertumoural heterogeneity between the two individuals, despite shared ligand–receptor pairs.

**FIGURE 4:**
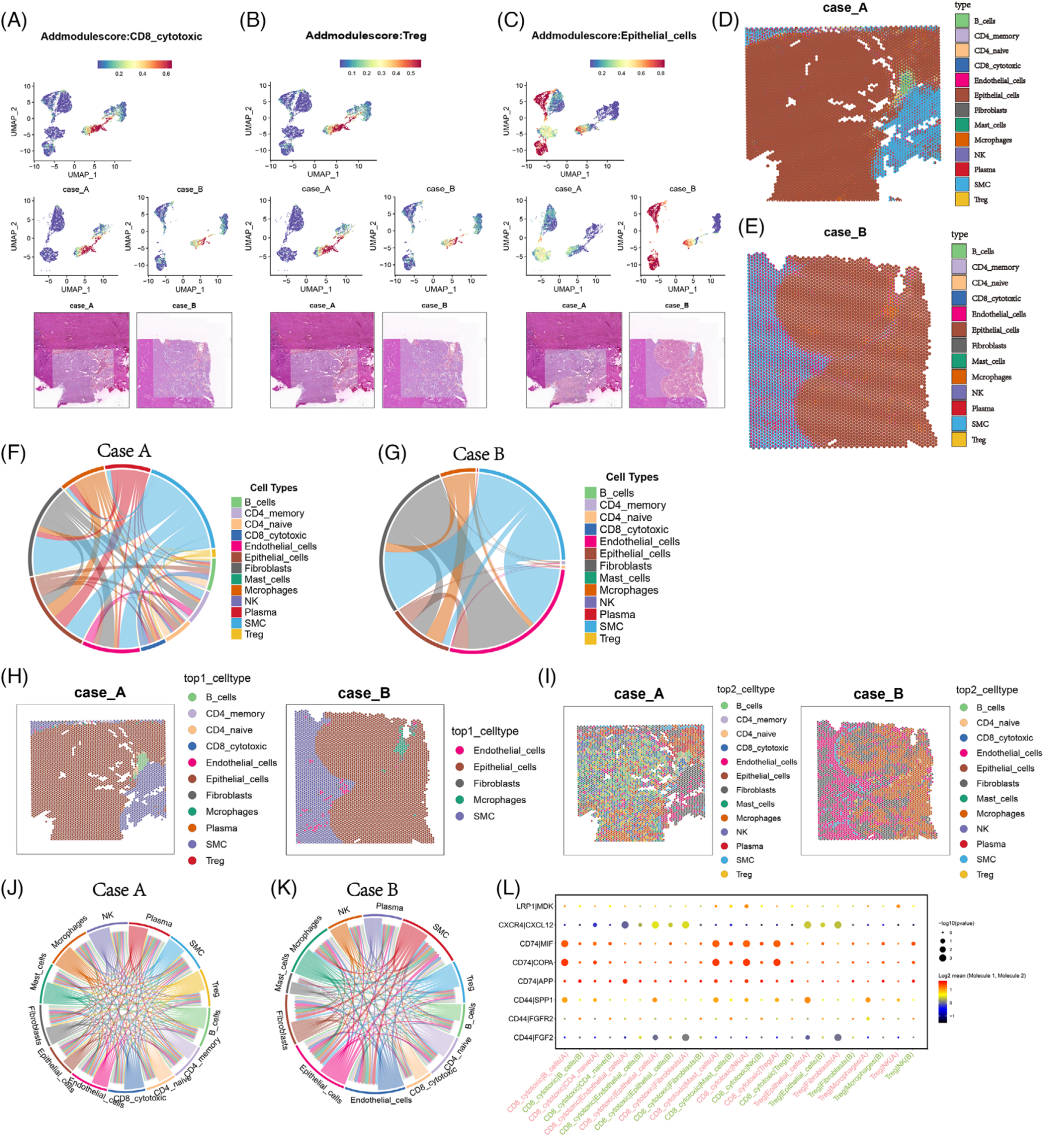
Cell distribution in the spatial transcriptome and cross‐talk of different types of cells within the TME. (A–C) Spatial feature plots of signature score of CD8 cytotoxic cells (A), Treg cells (B) and epithelial cells (C) in tissue sections. (D, E) Unbiased clustering of ST spots and define distinct cell types for each cluster from Case A (D) and Case B (E). (F, G) The circos plots illustrate the interactions between cell clusters in both Case A (F) and Case B (G), depicting the cell–cell communication for each case. (H, I) Spatial feature plots of the two spot patterns in Case A (H) and Case B (I) based on the Robust Cell Type Decomposition (RCTD) method. (J, K) Cell communication network in Case A (J) and Case B (K). The thickness of each string corresponds to the quantity of unique interaction pairs associated with individual cell clusters. (L) Bubble plots show ligand–receptor pairs between CD8 cytotoxic cells, Treg cells and other cells in Case A and Case B.

In conclusion, we systematically characterised variations in responsiveness between different patients with EC to anti‐PD‐1 therapy. This study identified heterogeneities in immune cells between patients responsive and non‐responsive to anti‐PD‐1 therapy. CD8^+^ cytotoxic cells and Tregs showed specific enrichment into patients responsive to anti‐PD‐1 therapy. Additionally, this work determined specific ligand–receptor interactions among CD8^+^ cytotoxic cells, Treg cells and other cells, which may affect the outcomes of tumour immunity. The obtained findings offer valuable insights into the potential therapeutic targets and biomarkers to elevate the efficacy of anti‐PD‐1 therapy. Although the present study provides initial insights into the characteristics associated with responses to anti‐PD‐1 treatment, we acknowledge the critical need for conducting studies involving larger cohorts.

## AUTHOR CONTRIBUTIONS

This study was conceived and designed by Xiaoping Wan and Yiran Li. Samples and clinical information were collected by Jian Huang. The experiments were conducted by Junfeng Chen and Yunfeng Song. Data analysis was performed by Jian Huang. The manuscript was written by Junfeng Chen and Yunfeng Song. Intellectual discussions and contributions to the manuscript content were provided by Junfeng Chen, Yunfeng Song, Xiaoping Wan and Yiran Li. This study was supervised by Junfeng Chen, Xiaoping Wan and Yiran Li. This study was reviewed and approved by all the authors.

## CONFLICT OF INTEREST STATEMENT

The authors declare no conflicts of interest.

## FUNDING INFORMATION

The study was supported by the National Natural Science Foundation of China (grant numbers 32270952, 32070583), the Shanghai Rising‐Star Program (grant number 22QC1400700), and the Shanghai Health System Outstanding Talents Program (grant number 20234Z0019).

## ETHICS STATEMENT

The Ethics Committee of Shanghai First Maternity and Infant Hospital, gave its approval to the project (ethics approval no. LS2101).

## Supporting information

Supporting information

Supporting information

Supporting information

Supporting information

Supporting information

## Data Availability

Single‐cell RNA sequencing and spatial transcriptomics data are accessible in the GEO database with accession code GSE251923.
