# Supplementary material for: Integrated single‐cell RNA sequencing and spatial transcriptomics analysis reveals the tumour microenvironment in patients with endometrial cancer responding to anti‐PD‐1 treatment
Source: Clin Transl Med. 2024 Apr 22;14(4):e1668. doi: 10.1002/ctm2.1668 (PMC11035376; doi:10.1002/ctm2.1668)
Supplement: Supplementary file 1 — Supporting information [file CTM2-14-e1668-s005.docx]

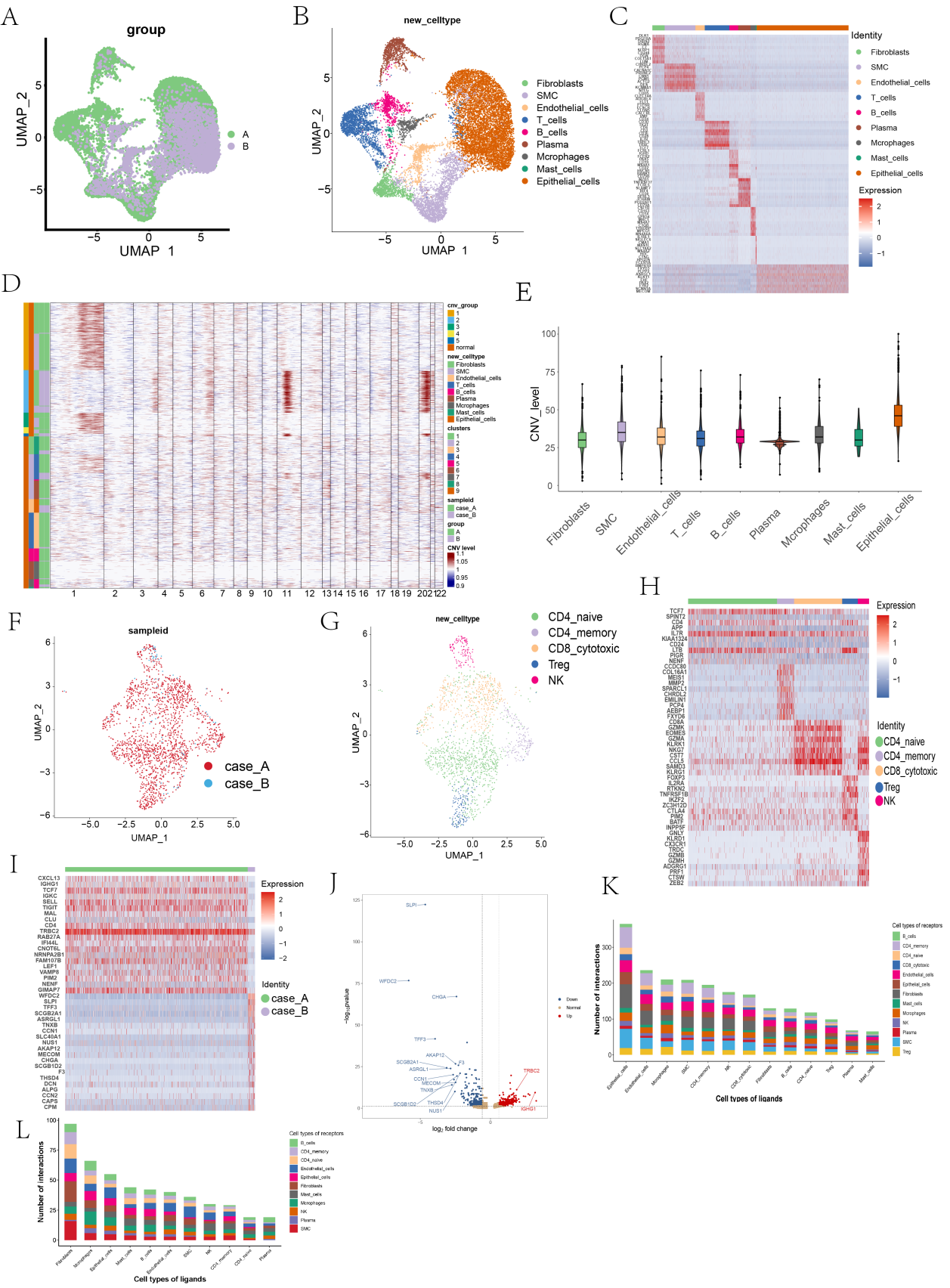


Figure S1 | Single-cell transcriptional profiles of endometrial cancer samples and transcriptional landscape and transcription factor regulons of T cells. (A) UMAP plot of main cell types in Case-A and Case-B from the snRNA-seq data. (B) UMAP plots display the expression of quintessential markers that are characteristic for each cell type. (C) The heatmap based on expression levels of top 10 marker genes in each cell type. (D) The heatmap shows large-scale CNVs of all cells. The red color represents high CNV level and blue represents low CNV level. (E) Violin plots show CNV levels among nine cell types. (F) UMAP plot of T cells in Case A and Case B from the snRNA-seq data. (G) UMAP visualizations reveal the expression patterns of key markers, uniquely representative of distinct T cell subpopulations. (H) The heatmap shows the top 10 significantly differentially expressed genes in each subset of T cells. (I) Heatmap of differentially expressed genes in each T subcluster. (J) Volcano plot showing the genes that are significantly differentially expressed genes in each subcluster of T cell. (K, L) Stacked bar charts illustrate the interaction count between different cell types and ligand-receptor pairs in Case-A (K) and Case-B (L).


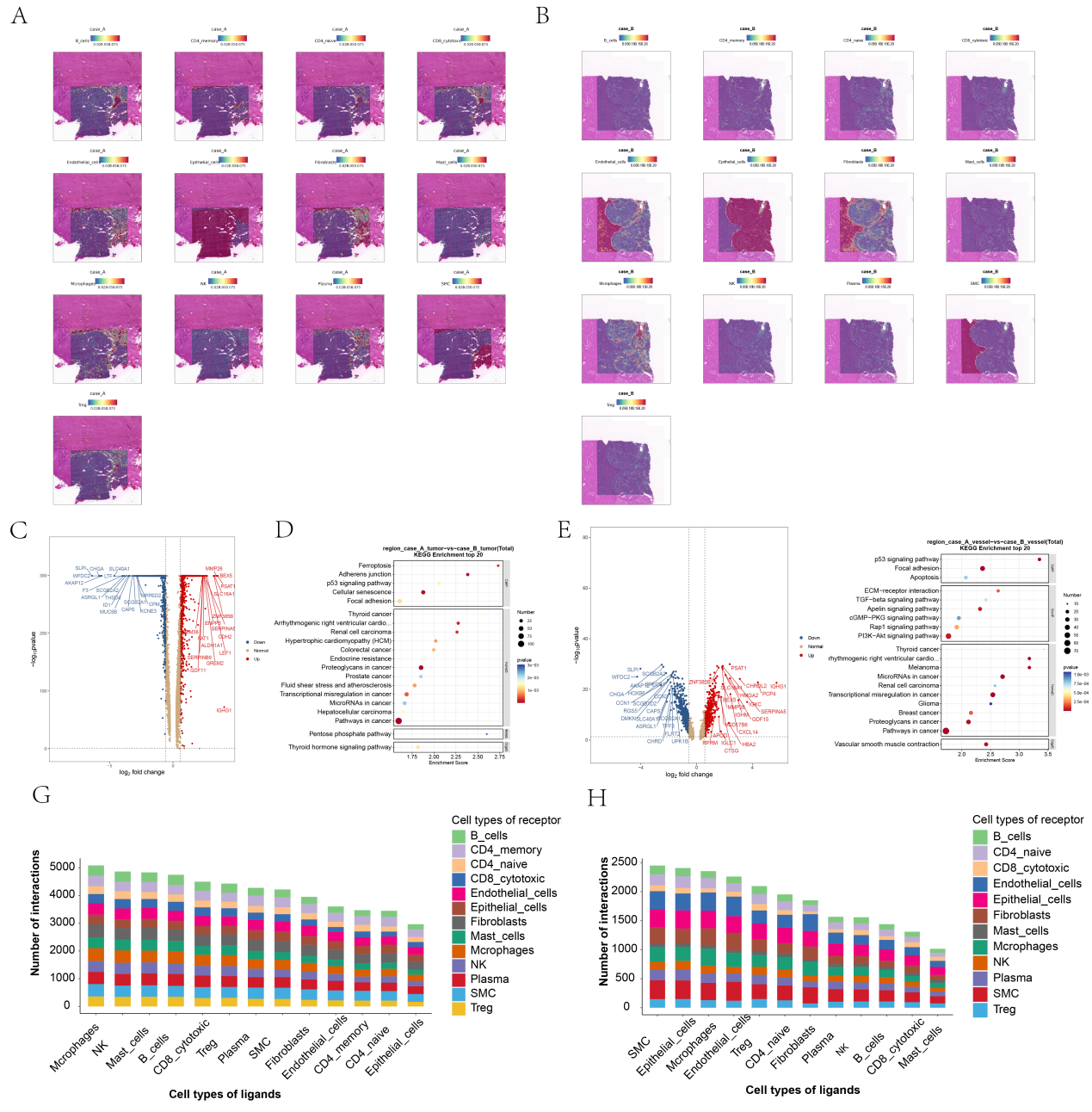


Figure S2 | Spatial profiles of cell type populations and multiple regulatory immune responses in the TME. (A, B) Spatial feature plots of the signature score of each type of cell in Case-A (A) and Case-B (B) tissue sections. (C, D) The volcano plot (C) and function enrichment (D) of differentially expressed genes in the tumor region of Case-A versus the tumor region of Case-B. (E, F) The volcano plot (E) and function enrichment (F) of differentially expressed genes in the vessel region of Case-A versus the vessel region of Case-B. (G, H) Stacked bar charts visually depict the interaction counts among various cell types and the ligand-receptor pairs in Case-A (G) and Case-B (H).
