# Supplementary figures and images for: Integrated single‐cell RNA sequencing and spatial transcriptomics analysis reveals the tumour microenvironment in patients with endometrial cancer responding to anti‐PD‐1 treatment
Source: Clin Transl Med. 2024 Apr 22;14(4):e1668. doi: 10.1002/ctm2.1668 (PMC11035376; doi:10.1002/ctm2.1668)

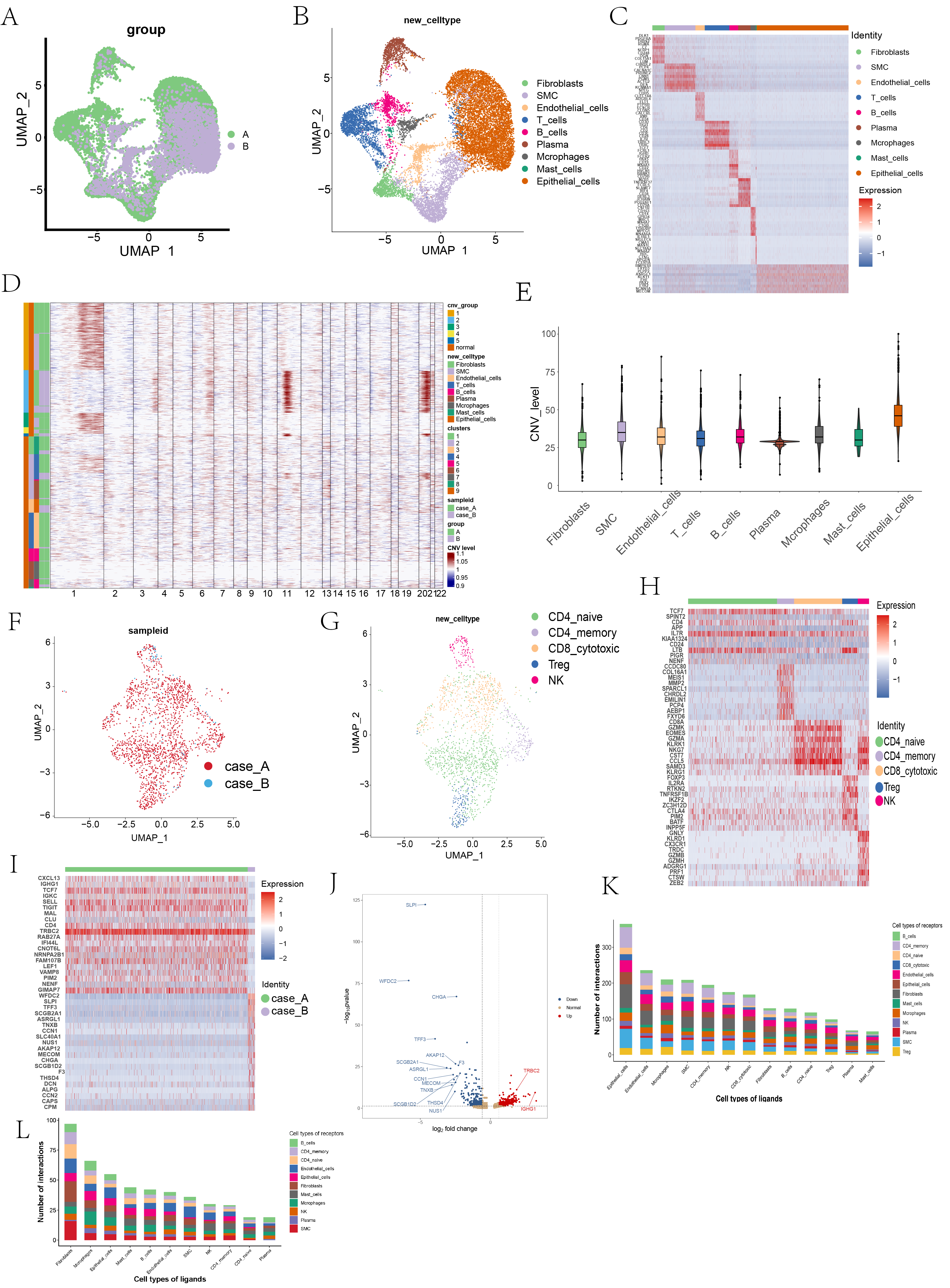

Supplement: Supplementary file 2 — Supporting information [file CTM2-14-e1668-s004.tif]

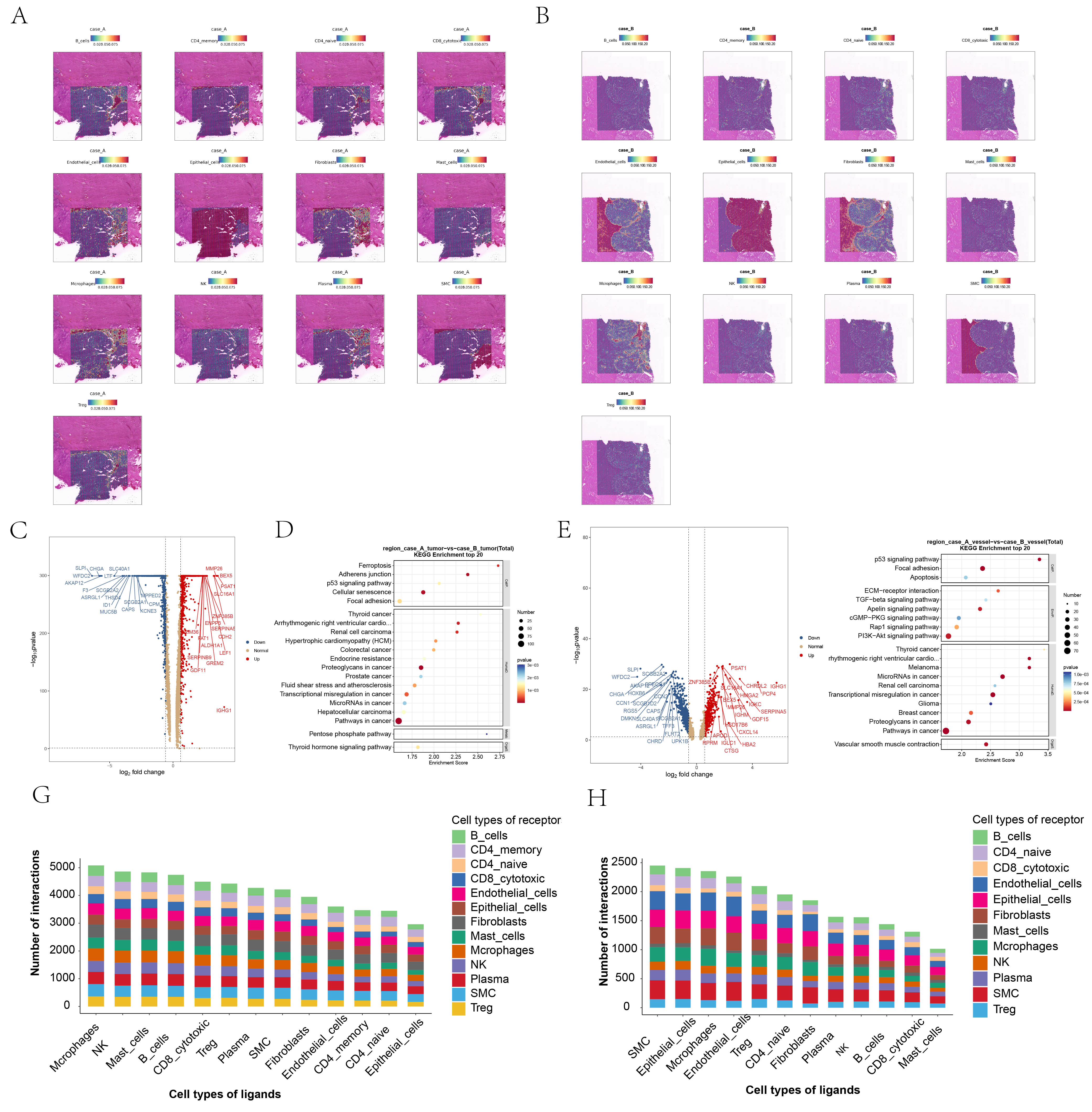

Supplement: Supplementary file 3 — Supporting information [file CTM2-14-e1668-s003.tif]
