# Supplementary material for: Integrated single‐cell RNA sequencing and spatial transcriptomics analysis reveals the tumour microenvironment in patients with endometrial cancer responding to anti‐PD‐1 treatment
Source: Clin Transl Med. 2024 Apr 22;14(4):e1668. doi: 10.1002/ctm2.1668 (PMC11035376; doi:10.1002/ctm2.1668)
Supplement: Supplementary file 4 — Supporting information [file CTM2-14-e1668-s001.docx]

| **Sample** | | Case-A | Case-B |
| --- | --- | --- | --- |
| **Status** | | Response | Non-response |
| **Age** | | 72 | 63 |
| **BMI** | | 20 | 22 |
| **Height** | | 158 | 158 |
| **Weight** | | 50 | 55 |
| **Organization type** | | Endometrial adenocarcinoma | Endometrial adenocarcinoma |
| **Stage** | | IA | IB |
| **FIGO** | | G3 | G1 |
| **Targeted therapy** | | Pembrolizumab +Lenvatinib | Pembrolizumab +Lenvatinib |
| **IHC staining** | **ER, PR** | + | +++ |
|  | **PTEN** | - | + |
|  | **Ki67** | 25%+ | 25%+ |
|  | **MLH1** | Deficient | Deficient |
|  | **PMS2** | Normal | Deficient |
|  | **MSH2** | Normal | Normal |
|  | **MSH6** | Normal | Normal |
| **Resource** | | Post-surgery | Post-surgery |
| **RECIST** | | PR | PD |

Table S1 Clinical and pathological features of two patients.

RECIST: Response Evaluation Criteria In Solid Tumors.

Partial Respond (PR).

Progressive Disease (PD).
