# Supplementary material for: Integrated single‐cell RNA sequencing and spatial transcriptomics analysis reveals the tumour microenvironment in patients with endometrial cancer responding to anti‐PD‐1 treatment
Source: Clin Transl Med. 2024 Apr 22;14(4):e1668. doi: 10.1002/ctm2.1668 (PMC11035376; doi:10.1002/ctm2.1668)
Supplement: Supplementary file 5 — Supporting information [file CTM2-14-e1668-s002.docx]

Integrated single-cell RNA sequencing and spatial transcriptomics analysis reveals the tumor microenvironment in endometrial cancer patients responding to anti-PD-1 treatment

Materials and Methods

**Patient samples**

Patient A is a 71-year-old patient who presented with postmenopausal vaginal bleeding and underwent hysteroscopic surgery at the hospital. Postoperative pathology revealed poorly differentiated adenocarcinoma, consistent with endometrioid carcinoma grade III. In the same month, she underwent laparoscopic staging surgery for endometrial cancer at our hospital. Postoperative pathology (E2021-12054) revealed endometrioid carcinoma grade III infiltrating the superficial myometrium of the uterus (<1/2), with the lower margin not reaching the internal of the cervix, and extensive intravascular tumor thrombus was observed. Five months later, she presented with vaginal bleeding again. Pelvic enhanced MRI suggested multiple solid nodules in the pelvic cavity, indicating postoperative recurrence of endometrial cancer. Patient B is a 63-year-old patient with no abnormal vaginal bleeding after menopause. Ultrasonography revealed slight thickening of the endometrium. Diagnosis by curettage pathology suggested complex atypical hyperplasia of the endometrium with local suspected carcinoma. She underwent total hysterectomy with bilateral salpingo-oophorectomy and pelvic lymph node dissection. Postoperative pathology (2017-06290) revealed endometrioid adenocarcinoma grade I, involving just the deep myometrium of the uterus (involving 1/2 of the myometrium), with the lower margin not reaching the internal of the cervix, and no intravascular tumor thrombus was observed. Fifty-three months later, she experienced persistent vaginal discharge. MRI suggested a residual solid mass in the vagina, indicating recurrence of endometrial cancer postoperatively. The FFPE samples used in this study were all based on postoperative pathology samples from the cases. Both cases A and B underwent a treatment switch to Lenvatinib/Pembrolizumab. Lenvatinib/Pembrolizumab was effective in Case-A but could not be continued after 13 cycles due to adverse events (hypertension). Case-A achieved a partial response after the 13-cycle treatment. By contrast, Case-B experienced disease progression with a re-enlargement of pelvic masses and lung nodules after completing seven cycles of treatment. The endometrial cancer samples were collected from all patients included in the study at Shanghai First Maternity and Infant Hospital, School of Medicine, Tongji University, Shanghai. Tables S1 included a set of sample comprehensive data. Every participant completed an informed consent form, which was approved by the Ethics Committee of Shanghai First Maternity and Infant Hospital.

**Single-cell dissociation**

The samples were immersed in the 4% formaldehyde fixative solution, following the precise protocol outlined in "Fixation of Cells & Nuclei for Chromium Fixed RNA Profiling". This fixation process was carried out using the Chromium Next GEM Single Cell Fixed RNA Sample Preparation Kit (10x Genomics PN-1000414), ensuring meticulous adherence to the established methodology.

**Single-cell RNA sequencing**

Depending on the presence and quantity of mixed samples, the corresponding 10xGenomics probe sets were introduced into the previously fixed specimens. Subsequently, an overnight incubation period was employed to facilitate the hybridization of these probes with their complementary target RNA molecules. Following the hybridization step, samples containing distinct probe barcodes were combined in equal numbers of cells/nuclei. Subsequent to the removal of unbound probes, Gel Bead-in-Emulsions (GEMs) were generated on the Chromium X platform using the Chromium Next GEM Chip Q Single Cell Kit and labeled Gel Beads. This was followed by primer hybridization for probe capture, sequence extension, and enzyme inactivation, all carried out through polymerase chain reaction (PCR). The hybridized products obtained from GEMs, after undergoing 8 rounds of pre-amplification, underwent adapter addition and fragment selection to yield the final sequencing library. Sequencing was performed using the PE-150 mode on the Illumina Nova platform.

**Quality control, dimension reduction and clustering analysis**

Using the official 10x Genomics software, Cell Ranger, sample quality control was conducted. This software internally integrates STAR, which aligns reads to a reference genome. This alignment process provided essential quality control metrics from the raw data, including the number of high-quality cells, gene counts, and genome alignment rates. These metrics were instrumental in assessing the quality of each sample. Given the presence of batch effects among different samples in our project, a strategic approach was employed. Firstly, we utilized the Mutual Nearest Neighbors (MNN) dimensionality reduction algorithm to effectively remove batch effects [1]. Subsequently, building upon the MNN-based dimensionality reduction results, we applied the Uniform Manifold Approximation and Projection (UMAP) algorithm to visualize single-cell clustering [2]. Our clustering analysis was underpinned by the Shared Nearest Neighbor (SNN) algorithm, resulting in the optimal cellular subgroups.

**Unsupervised clustering and annotation**

According to the marked genes of each cell cluster, these cells were divided into many distinct cell clusters, including epithelial cells, endothelial cells, fibroblasts, NK/T cells, B cells, plasma cells, macrophages, SMCs and mast cells. To discern distinct subgroups within the primary cell types identified, the secondary UMAP reduction process was implemented. Clusters were designated based on the genes that were most prominently expressed within each group [3].

**Gene set variation analysis**

To assess the differential signaling pathway activation across various cell clusters, Gene Set Variation Analysis (GSVA) was utilized for quantifying gene set enrichment scores [4]. This process entailed executing the GSVA function on each individual cell, employing the single sample gene set enrichment analysis (ssGSEA) method for determining the enrichment scores of specific gene sets [5]. Subsequently, the limma package was applied to contrast the enrichment scores of each gene set across different cell clusters. For each cluster, the five most significantly up-regulated gene sets were identified. These scores were then normalized across the clusters and visually represented in a heatmap format. This approach ensured a comprehensive and comparative analysis of pathway enrichment among distinct cellular populations. To compute the infiltration score of specific subsets within the bulk RNA-sequencing data, we utilized the single sample gene set enrichment analysis [5].

**SCENIC analysis**

Transcription factors (TFs) orchestrate a complex network of gene regulation through their downstream targets, shaping and sustaining cellular identity [6]. For the analysis of transcription factor activity, we employed the SCENIC package, version 1.1.2.1, a tool specifically designed for inferring and clustering single-cell regulatory networks [6]. In our approach, we developed 'super cells' by amalgamating the data from every 20 individual cells within each cluster. This strategy was employed to minimize the consumption of computational resources. The mean values of normalized counts of 20 single cells were calculated as the raw input data of SCENIC. This method examines the functionality of regulons, each of which encompasses a TF and its associated target genes. Modules within the regulon network were identified through an analysis of regulon crosstalk, employing the connection specificity index (CSI) [7]. This approach ranks the significance of each regulon, effectively reducing the effects of nonspecific interactions in the network. To identify the differentially activated TFs in each subcluster, we employed the Wilcoxon rank sum test. We set stringent criteria, considering TFs with a log fold change > 0.1 and an adjusted *P*-value < 0.05 as significantly altered.

**Cell communication analysis**

Cell communication analysis was conducted utilizing CellPhoneDB, a publicly accessible repository containing information on receptor-ligand interactions [7]. The normalization of the cell matrix was accomplished through Seurat Normalization. Significance in cell communication was determined by assessing p-values (p < 0.05), along with the calculation of significant means based on the interactions. Analysis of ligand-receptor interactions within the scRNA-seq data was conducted using the iTalk R package [8]. Additionally, Giotto was employed to identify interactions in the spatial transcriptomics data.

**Spatial transcriptomics**

Tissue samples, following embedding in optimal cutting temperature compound, were sectioned into 10μm slices. Subsequently, Spatial Transcriptomics were performed using a Visium Spatial Gene Expression Kit from 10x Genomics. Initially, we optimized tumor tissue permeability conditions using a kit for a duration of few minutes, followed by subsequent staining with hematoxylin and eosin (H&E). Following imaging using a Leica microscope at 20× magnification, subsequent ST processing was conducted. Only one slice was ultimately used for each sample due to the analysis of the final spatial transcriptome experiment. The ROI means regions of interest. All the ROIs were evaluated and confirmed by two pathologists from Shanghai First Maternity and Infant Hospital (Shanghai, China). The resulting data provide whole transcriptome quantification for each ROI that can be compared across regions.

**10x Visium spatial RNA-seq data preprocessing**

The processing of Visium spatial transcriptome sequencing data and bright-field microscopy slice images was carried out using the official 10x Genomics software, Space Ranger. This workflow involved the identification of capture regions within the tissue on the chip. Based on the spatial barcode information, the software distinguished and segregated reads for each spot, enabling the computation of key metrics, including the total number of spots, reads per spot, detected gene counts, and UMIs. These metrics played a crucial role in the quality assessment of the samples. Furthermore, we integrated STAR software for alignment analysis against a reference genome, resulting in the generation of a gene-spot matrix. This matrix was then utilized for subsequent gene expression analysis.

**Multiplex Immunofluorescence**

The immunofluorescence assay conducted on tumor tissues followed a previously established method [9]. Formalin-fixed, paraffin-embedded (FFPE) tissue sections were initially melted at 60°C for 120 minutes. This step was succeeded by a dewaxing process involving sequential immersion in xylene (three times, 5 minutes each) and 100% ethanol (three times, 10 minutes each), concluding with a final treatment in 95% alcohol for 10 minutes. Antigen retrieval was conducted by heating the samples at 98 °C for 10 minutes using a 10 mM citrate buffer at pH 6.0. Following this, endogenous peroxidase activity was suppressed by treating the sections with a 3% hydrogen peroxide solution for a duration of 10 minutes. Subsequently, the tissue sections were allowed to cool for 30 minutes. This was followed by three successive washes in 0.02% tris-buffered saline–Tween 20 (TBST), each lasting 5 minutes with gentle agitation. Sections were incubated for overnight at 4 °C with anti-CD8, anti-CD20, anti-CD56 and anti-CD68. On the subsequent day, the sections underwent triple rinsing with PBS mixed with 0.1% Tween-20 for 15 minutes per cycle on an orbital shaker. Post-primary antibody incubation, they were then exposed for two hours at ambient temperature to secondary antibodies, which were diluted in a solution of 1% BSA in PBS. Hoechst 33342 (Invitrogen) was used to stain nuclei, and the resulting immunofluorescence images were captured utilizing the Leica laser scanning confocal microscope.

Reference

1. Haghverdi, L., et al., *Batch effects in single-cell RNA-sequencing data are corrected by matching mutual nearest neighbors.* Nat Biotechnol, 2018. **36**(5): p. 421-427.

2. Becht, E., et al., *Dimensionality reduction for visualizing single-cell data using UMAP.* Nat Biotechnol, 2018.

3. Zhang, Y. and H. Kiryu, *MODEC: an unsupervised clustering method integrating omics data for identifying cancer subtypes.* Brief Bioinform, 2022. **23**(6).

4. Hänzelmann, S., R. Castelo, and J. Guinney, *GSVA: gene set variation analysis for microarray and RNA-seq data.* BMC Bioinformatics, 2013. **14**: p. 7.

5. Liu, Y., et al., *Single-cell analysis reveals immune landscape in kidneys of patients with chronic transplant rejection.* Theranostics, 2020. **10**(19): p. 8851-8862.

6. Aibar, S., et al., *SCENIC: single-cell regulatory network inference and clustering.* Nat Methods, 2017. **14**(11): p. 1083-1086.

7. Efremova, M., et al., *CellPhoneDB: inferring cell-cell communication from combined expression of multi-subunit ligand-receptor complexes.* Nat Protoc, 2020. **15**(4): p. 1484-1506.

8. Xie, Z., X. Li, and A. Mora, *A Comparison of Cell-Cell Interaction Prediction Tools Based on scRNA-seq Data.* Biomolecules, 2023. **13**(8).

9. Tan, W.C.C., et al., *Overview of multiplex immunohistochemistry/immunofluorescence techniques in the era of cancer immunotherapy.* Cancer Commun (Lond), 2020. **40**(4): p. 135-153.
